# Mitochondrial dysfunction in cellular senescence: a bridge to neurodegenerative disease

**DOI:** 10.1038/s41514-025-00291-4

**Published:** 2025-12-16

**Authors:** Adam J. Hruby, Ryo Higuchi-Sanabria

**Affiliations:** Leonard Davis School of Gerontology, University of Southern California, Los Angeles, CA USA

**Keywords:** Cell biology, Neuroscience, Physiology

## Abstract

Senescent cells, characterized by a state of irreversible proliferative arrest and inflammatory profile, have emerged as drivers of age-related decline. Growing evidence suggests that alterations in mitochondrial function and morphology play a key role in the induction and maintenance of senescence, as well as in promotion of the proinflammatory senescence-associated secretory phenotype (SASP). In this review, we seek to survey the relationship between mitochondrial dysfunction and senescence, focusing on the consequences of changes in oxidative phosphorylation efficiency, calcium handling, mitochondrial metabolites, mitochondrial dynamics and quality control, and release of damage-associated molecular patterns. We first describe these changes before illustrating the pathways and mechanisms through which mitochondrial dysfunction results in cell cycle arrest and the SASP. Lastly, we showcase evidence relating cellular senescence to neurodegenerative disease and propose that mitochondrial dysfunction may act as a bridge between the two.

## Introduction

The nature and cause of aging have long remained a topic of considerable debate, despite its widespread appearance across species. One of the most prevalent and influential theories to explain aging was proposed by Denham Harman in 1955 as the free radical theory of aging^1^. In it, Harman postulates that aerobic respiration could produce reactive oxygen species (ROS) that contain an unpaired electron, which can damage molecules within cells and impair their function. The accumulation of oxidative damage over time leads to a decline in tissue and organ function, ultimately resulting in the aging phenotype. Nearly two decades later, Harman revised his theory to include the role of mitochondria, the organelles responsible for cellular respiration, proposing the mitochondrial free radical theory of aging (MFRTA)^2^. The MFRTA states that, by virtue of their role in respiration, mitochondria are responsible for producing damaging ROS. Later refinement of the theory proposed a positive feedback loop or “vicious cycle” in which damage to mitochondria and mitochondrial DNA (mtDNA) by ROS would promote the production of further ROS, causing a decline in mitochondrial function and a cascade of damage to cells, tissues, organs, and the organism^3^.

Efforts to test the MFRTA using various methods have yielded mixed and sometimes conflicting results. An assertion arising from the MFRTA is that the mitochondria of longer-lived species should produce fewer ROS than those of shorter-lived species. In general, this association appears to hold true, at least in regards to superoxide and hydrogen peroxide (H_2_O_2_) production^4–6^. However, species that do not hold to this trend, such as the naked mole rat and questions regarding the validity of the methods used to measure H_2_O_2_ production have called the accuracy of these findings into question^5,7^. A more direct way of testing the MFRTA is through the manipulation of the cell’s ability to combat the effects of ROS via free radical-quenching antioxidant molecules or enzymes, which should presumably reduce oxidative damage and thus extend lifespan, while decreasing these defenses should decrease lifespan. Antioxidant supplements have been tested in several different model organisms, including *Caenorhabditis elegans*, *Drosophila melanogaster*, and *Mus musculus,* with mixed results. In invertebrates, many antioxidants tested do exert a positive influence on lifespan^6,8^; again, with exceptions. For example, chronic administration of dietary thiols, important cellular antioxidants, decreases lifespan in *C. elegans*^9^. In addition, exposure of *C. elegans* to ROS-generating compounds can, in fact, increase lifespan^6,10^, likely via activation of the unfolded protein response of the mitochondria^11^. Results of antioxidant supplementation in mice are even more heterogenous^8,12,13^. Notably, no extension of lifespan occurs with administration of coenzyme Q_10_, a mitochondrial antioxidant^14^ and no consistent effect on lifespan is observed either with overexpression or knockouts of several antioxidant enzymes^6,12,15–17^. Likely, these inconsistent results are driven by the complex roles of ROS as a signaling molecule in diverse pathways, some of which can have beneficial effects^18^.

The ambiguous results produced from efforts to validate the MFRTA have now shifted the field to characterize oxidative damage from ROS as being one of many sources of damage^19^ that contribute to a diverse array of interconnected aging hallmarks^20^, rather than being a sole, primary driver of aging. However, progress in mitochondrial research made since the inception of the MFRTA has revealed mitochondria as dynamic players in many cellular processes. Mitochondria undergo a variety of changes that can have both beneficial and detrimental effects on cellular processes, influencing metabolism, signaling, and cell fate decisions in complex ways. The regulation of mitochondrial function is thus central to cellular function, organismal health, aging, and disease. Given the breadth of this topic, we focus here on a specific area of interest to the aging field: how mitochondrial alterations contribute to cellular senescence, another key hallmark and driver of aging. Here, we will review the changes that mitochondria undergo in senescent cells and describe the role this plays in driving senescence-related pathology, ultimately explaining how these changes manifest as a driver of age-related neurodegenerative disease.

## Cellular senescence and aging

Cellular senescence, first described in the early 1960s^21^, is a state of irreversible cell cycle arrest that results in widespread changes to gene expression and cell morphology^22,23^. Senescent cells play important physiological roles in development, wound healing, and cancer suppression, but contribute to age-related pathology in later life^24^. Senescence can be induced by a wide variety of means, including telomere shortening (replicative senescence (RS)), activation of oncogenes (oncogene-induced senescence (OIS)), and exposure to genotoxic agents, such as radiation (stress-induced senescence (SIS))^23^. These cause DNA damage, leading to the recruitment of protein kinases, such as ataxia-telangiectasia mutated (ATM) or ataxia telangiectasia and Rad3-related (ATR) and initiation of the DNA damage response (DDR)^25^. This ultimately induces a stable cell-cycle arrest through the cyclin-dependent kinase inhibitors p16 and p21 via the tumor-suppressive transcriptional regulators pRB and p53, respectively^23^. In addition to cell cycle arrest, senescent cells undergo a change in morphology to become large and flat, increase lysosomal β-D-galactosidase activity termed senescence-associated β-galactosidase (SA-β-gal)^26,27^, and display resistance to apoptosis^28^. Increases in markers of DNA damage, including γH2AX and 53BP1, changes in heterochromatin, loss of Lamin B1, relocation of high mobility group box 1 (HMGB1) from the nucleus to cytoplasm, and fragments of chromatin present in the cytoplasm^29,30^ are also observed in senescence. A combination of these and other phenotypic changes is used to identify senescent cells in culture and tissues.

The lack of a definitive senescence marker has led to some controversy over what constitutes a senescent cell. For example, in response to immune stimulation, macrophages express p16 and SA-β-gal, which reflect an activated state and not senescence^31^. In addition, single-cell and single-nucleus transcriptomic approaches have revealed the heterogeneous nature of senescent cells^32^. Single-cell RNA-seq of RS, irradiation-induced senescence, and etoposide-induced senescence revealed similarities in the transcriptome between both SIS models, but significant variation from RS cells^33^. Furthermore, several distinct clusters emerged from this analysis, including growth-arrested cells, which did not exhibit other senescent phenotypes, a group displaying the traditional senescence markers, and another senescent group associated with changes in ECM proteins, anti-apoptotic pathways, and long non-coding RNAs. These studies demonstrate that cellular senescence cannot be viewed as a monolith but instead manifests in diverse ways, highlighting the necessity of testing for multiple markers of senescence.

One significant pathological aspect of senescent cells is the secretion of signaling factors, including cytokines, chemokines, interleukins, growth factors, proteases, insoluble proteins, and extracellular matrix (ECM) components^34^. The central regulator of these inflammatory factors is nuclear factor kappa-light-chain-enhancer of activated B cells (NF-κB), which is activated as a result of DNA damage^35^. Together, these factors make up what is known as the senescence-associated secretory phenotype (SASP) and are believed to be one of the primary ways in which senescent cells contribute to tissue dysfunction and aging.

Current interest in cellular senescence largely focuses on the role senescent cells play in the aging process. Senescent cells accumulate with age in a number of species across a wide variety of tissues^36–39^, although the exact proportion of senescent cells in aged tissues remains controversial due to a lack of a single biomarker for senescence. Senescent cell accumulation is believed to be due to a decline in immune surveillance with age, as impairment to immune cell function accelerates senescent cell burden^40,41^. In tissue, senescent cells contribute to a proinflammatory microenvironment via the SASP^42^, which can act in both an autocrine and paracrine manner^43,44^, reinforcing senescence as well as inducing senescence in neighboring cells^45,46^. Thus, recent work has focused on demonstrating that elimination of senescent cells—either through genetic means or the use of senolytic drugs that selectively induce apoptosis in senescent cells—can extend healthspan and lifespan^47–49^, and a multitude of clinical trials targeting diverse conditions using senolytics are underway^50,51^. Like any other therapeutic, it will be important to consider possible side effects when administering senolytic treatments, particularly in light of the role senescent cells play in wound healing^52^ and liver regeneration^53^. Nevertheless, by targeting the largest risk factor for the diseases of late life, aging itself, senotherapeutics hold promise for the future of medicine, spurring the need to further understand the mechanisms that underlie the senescent phenotype. As investigations into these mechanisms have progressed, mitochondria have emerged as central players in both senescence induction and the SASP.

## Mitochondrial dysfunction in cellular senescence

**Fig. 1:**
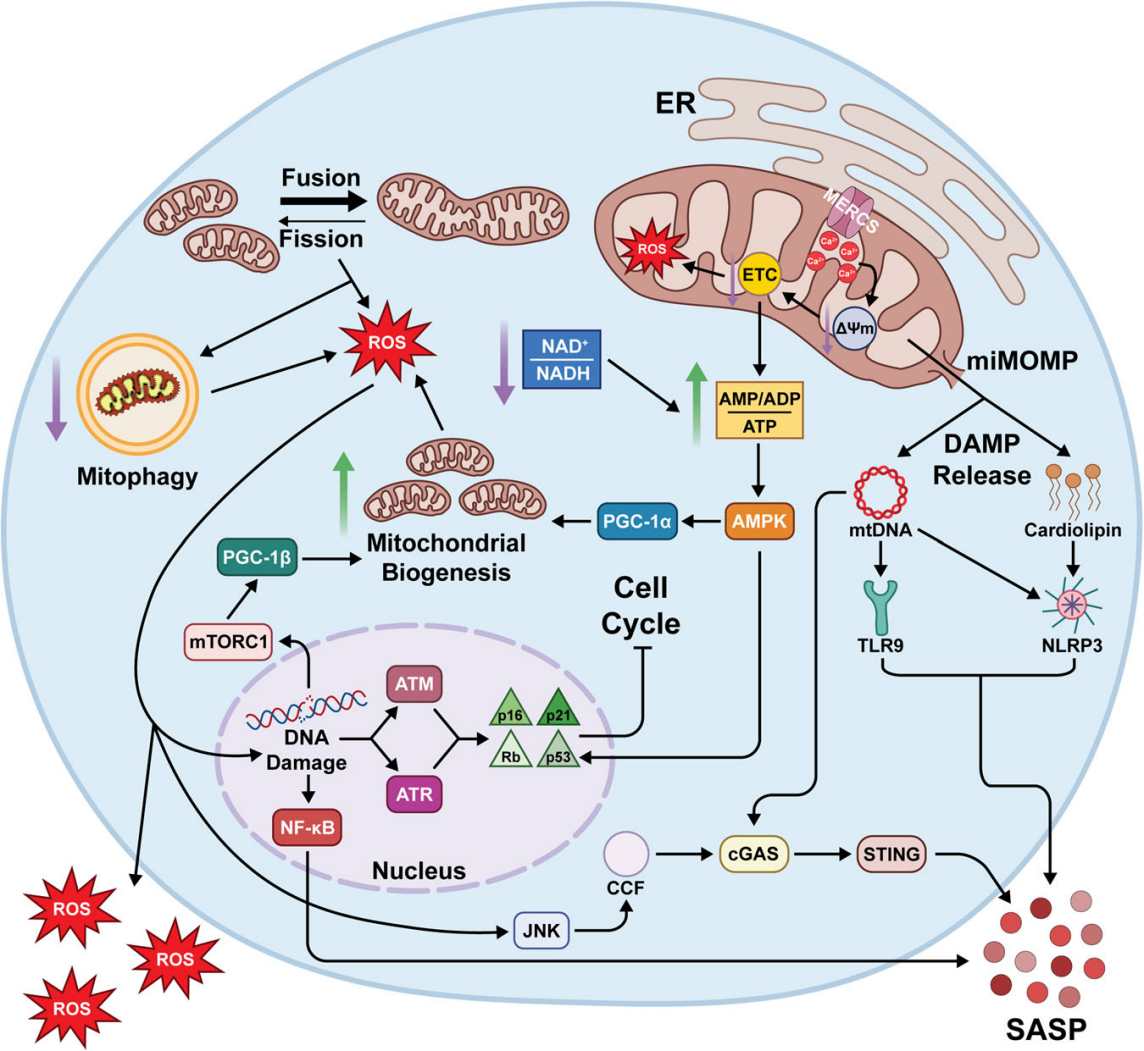
Mitochondrial dysfunction during cellular senescence aids proliferative arrest and enhances the SASP. Mitochondrial membrane potential (ΔΨm) decreases during senescence as a result of accumulating calcium from the endoplasmic reticulum (ER) through mitochondria-ER contact sites (MERCS) and increased proton leakage. These and other changes decrease electron transport chain (ETC) efficiency, increasing reactive oxygen species (ROS) production and decreasing ATP production. An imbalance in the mitochondrial metabolites NAD^+^/NADH exacerbates declining ATP levels, promoting activation of AMPK. In addition to supporting cell cycle arrest, activated AMPK promotes mitochondrial biogenesis. Mitochondrial biogenesis and additional changes to morphological regulation, including increased mitochondrial fusion and decreased mitophagy, further aid ROS production. DNA damage caused by ROS enforces cell cycle arrest and promotes the SASP. In addition, ROS activates JNK, which promotes the formation of cytosolic chromatin fragments (CCFs), activating the cytosolic DNA sensor cGAS, which, through STING activation, enhances SASP production. Minority mitochondrial outer membrane permeabilization (miMOMP) releases inflammatory DAMPs, including mtDNA and cardiolipin, which further enhances the SASP.

Mitochondria of senescent cells exhibit a myriad of changes in activity and morphology (Fig. 1), many of them similar to those that drive aging pathology^20^. Here, we describe these changes, which are consistent with mitochondrial dysfunction. Classically, the definition of mitochondrial dysfunction is a reduction in mitochondrial membrane potential and a corresponding loss in respiratory capacity per mitochondrion when at a steady state. We expand this definition beyond bioenergetics to encompass impairment in the maintenance of mitochondrial form and any of its multifaceted functions within the cell. Further, we explore studies which demonstrate that mitochondrial dysfunction is causative in senescence induction and that targeting mitochondria has therapeutic potential. Mitochondrial-based interventions from studies that demonstrate anti-senescence effects are summarized in Table 1.

**Table 1:** Mitochondrial-targeted anti-senescence interventions and their biological outcomes

Mitochondrial dysfunction	Anti-senescence intervention	Mitochondrial target	Model	Biological outcome	Reference
ETC	Temporal antimycin A treatment	Transient complex III inhibition	SIS human fetal lung fibroblasts (MRC-5)	• Reduced SA-β-gal activity • Increased Ki-67-positive cells • Reduced intracellular ROS • Increased ΔΨm • Decreased ATP levels • Increased mitochondrial biogenesis • Reduced mitophagy	^75^
ROS	Mitoquinone mesylate (MitoQ)	Mitochondrial antioxidant	Doxorubicin-induced senescent human umbilical vein endothelial cells (HUVECs)	• Increased proliferation • Reduced p16 • Reduced mitochondrial superoxide • Reduced IL-8, IL-6, and MCP-1 • Reduced 53BP1 DNA damage marker	^87^
	Superoxide dismutase 3 overexpression	Antioxidant	Human fetal lung fibroblasts (MRC-5)	• Increased replicative lifespan under normoxia and hyperoxia • Decreased intracellular peroxides • Slowed telomere shortening under normoxia and hyperoxia	^88^
	α-phenyl-t-butyl-nitrone	Antioxidant	Human fetal lung fibroblasts (MRC-5)	• Increased replicative lifespan • Decreased intracellular peroxides under normoxia, hyperoxia, and in SIS • Slowed telomere shortening	^89^
	Carnosine	Antioxidant	Human foreskin fibroblasts (HFF-1); human fetal lung fibroblasts (MRC-5)	• Increased replicative lifespan • Maintenance of non-senescent morphology	^91^
	Aminoguanidine	Antioxidant	Progeroid mice-derived dermal fibroblasts	• Increased replicative lifespan • Maintenance of non-senescent morphology • Reduced lipid peroxides	^92^
			Human fetal lung diploid fibroblasts (2BS)	• Increased replicative lifespan • Slowed telomere shortening • Maintenance of non-senescent morphology • Reduced DNA damage after H_2_O_2_ exposure	^93^
ROS/Metabolism	Nicotinamide	Antioxidant, increased NAD^+^ levels, increased sirtuin activity	Human foreskin fibroblasts	• Increased replicative lifespan • Reduced SA-β-gal activity • Decreased p53 and p21 protein levels • Decreased ROS • Slowed telomere shortening • Decreased ATP levels • Increased ΔΨm	^94^
Metabolism	Cyanidin-3-O-glucoside or CD38 siRNA	Inhibition or knockdown of CD38 increasing the NAD^+^/NADH ratio leading to SIRT6 activation	D-galactose-induced senescent rat myocardial cells (H92C)	• Reduced SA-β-gal activity • Increased proliferation • Increased telomerase reverse transcriptase mRNA levels • Decreased ROS • Increased NAD^+^/NADH ratio • Decreased CD38 mRNA and protein levels • Increased SIRT6 mRNA and protein levels	^372^
	Cyanidin-3-O-glucoside	Inhibition of CD38 increasing the NAD^+^/NADH ratio leading to SIRT6 activation	Myocardial tissue of 16-week-old D-galactose acute aging mouse model	• Increased NAD^+^/NADH ratio • Decreased CD38 mRNA and protein levels • Increased SIRT6 mRNA and protein levels • Decreased collagen • Improved fiber morphology • Decreased SASP factors (IL-1α, IL-1β, IL-6, IL-10, IL-17A, TNF-α) in peripheral blood • Improved oxygen consumption	^372^
	Metformin	AMPK activation inducing autophagy	Hydrogen peroxide-induced senescent human lens epithelial cells (HLE-B3)	• Reduced p16 mRNA levels • Reduced p21 mRNA and protein levels • Reduced p53 protein levels • Reduced SA-β-gal activity • Reduced IL-6 and IL-8 mRNA levels • Improved autophagic flux • mTOR inhibition	^167^
	Metformin or berberine	AMPK activation increasing NAD^+^/NADH ratio through NAMPT expression and activating SIRT1 and autophagy	Hydrogen peroxide-induced senescent mouse embryonic fibroblasts (NIH/3T3)	• Reduced SA-β-gal activity • Increased NAMPT mRNA and protein levels • Increased NAD^+^/NADH ratio • Increased SIRT1 activity • Improved autophagic flux	^168^
	Licochalcone D	AMPK activation inducing autophagy	Oxidative stress-induced senescent human bone marrow-mesenchymal stem cells (hBM-MSCs)	• Reduced p16, p21, and p53 protein levels • Reduced SA-β-gal activity • Increased autophagic flux	^169^
		AMPK activation	17-week-old D-galactose acute aging mouse model	• In heart and hippocampus: reduced p21 and p53 protein levels, increased phosphorylated AMPK • In heart: increased autophagy markers • In hippocampus: reduced receptor for advanced glycation end-products expression	^169^
	Nifedipine	Calcium channel block leading to AMPK activation and increased autophagy	Hydrogen peroxide-induced senescent primary rat vascular smooth muscle cells (VSMCs)	• Reduced p53 and p21 protein levels • Reduced SA-β-gal activity • Decreased intracellular calcium • mTOR inhibition • Increased autophagic flux	^170^
Increased mitochondrial biogenesis/ROS	Rapamycin	mTORC1 inhibition-mediated reduction in PGC-1β-dependent mitochondrial biogenesis	Irradiation-induced senescent human fetal lung fibroblasts (MRC-5)	• Reduced SA-β-gal activity • Decreased p21 mRNA and protein levels • Reduced ROS, reduced γH2AX DNA damage marker • Reduced IL-6 mRNA	^98^
			15 to 16-month-old C57/BL6 mouse liver	• Reduced SA-β-gal levels • Reduced p21 protein levels • Reduced telomere-associated foci • Reduced SASP (CXCL-1 and inhibin A mRNA levels)	^98^
Increased mitochondrial biogenesis	Resveratrol, salidroside	Increased mitochondrial biogenesis without concomitant ROS increase via SIRT1 upregulation	Replicative senescent human fetal lung fibroblasts (MRC-5)	• Reduced SA-β-gal activity • Reduced intracellular ROS • Increased ΔΨm • Increased ATP levels	^122^
Mitochondrial elongation	FIS1 upregulation	Increased mitochondrial fission	RNAi-mediated FIS1-depleted cervical cancer cells (HeLa)	• Reduced SA-β-gal activity	^123^
Mitochondrial elongation and impaired mitophagy	siRNA-mediated p53 knockdown	Increased mitochondrial fission via decreased expression of OPA1 and increased expression and phosphorylation of DRP1	Calcified mouse vascular smooth muscle cells (VSMCs)	• Reduced p53 and p21 protein levels • Reduced SA-β-gal activity • Increased ΔΨm • Reduced mitochondrial ROS	^373^
Impaired mitophagy	Rapamycin, nicotinamide (NAM), or nicotinamide riboside (NR)	PINK1/Parkin-dependent mitophagy activation	Irradiation-induced senescent primary neonatal human diploid fibroblasts (HDFs)	• Reduced p16 and p21 levels • SA-β-gal activity • Reduced cell size • Did not rescue proliferation or suppress IL-6 and IL-8 mRNA levels	^101^
	IGF-1	Mitophagy upregulation via the NRF2/SIRT3 pathway activation	Replicative senescent mouse vascular smooth muscle cells (VSMCs)	• Reduced p16 and p21 protein levels • Reduced SA-β-gal activity • Increased ΔΨm • Maintained telomere length • Reduced mtDNA damage	^115^
Generalized mitochondrial dysfunction	Methylene blue	Redox cycling	Human fetal lung fibroblasts (IMR90)	• Extended replicative lifespan • Extended replicative lifespan under exposure to hydrogen peroxide or cadmium • Increased oxygen consumption • Increased complex IV expression • Increased heme synthesis • Increased NADH oxidation	^194^
			Human fetal lung fibroblasts (IMR90)	• Increased PGC-1α expression • Increased NAD^+^/NADH ratio • Increased complex IV activity • Decreased intracellular oxidants • Extended replicative lifespan • Slowed telomere erosion	^195^
			Human primary skin fibroblasts	• Decreased mitochondrial ROS • Decreased p16 levels • Decreased SA-β-gal activity • Increased expression of NRF2 pathway • Increased proliferation	^90^
			Cholestatic livers of Sprague-Dawley rats (bile duct ligation model)	• Mitigated splenomegaly and hepatomegaly • Increased ΔΨm • Decreased mitochondrial lipid peroxidation • Increased mitochondrial dehydrogenase activity • Increased ATP	^196^
			C57/BL6 mice multiple sclerosis model (cuprizone-fed)	• Decreased demyelinated neurons in the corpus callosum • Increased locomotor activity • Increased ΔΨm • Decreased mitochondrial lipid peroxidation • Increased mitochondrial dehydrogenase activity • Increased ATP levels	^197^
	Klotho upregulation	Senescence-associated mitochondrial dysfunction	Unilateral ischemia-reperfusion (UIRI) mouse kidney	• Increased mitochondrial mass • Decreased mitochondrial ROS • Improved mitochondrial morphology • Reduced p16 protein levels • Reduced SA-β-gal activity • Reduced p19 and γH2AX mRNA and protein levels	^201^
			Prolong cultured human cortical brain organoids	• Reduced p16 mRNA levels • Reduced p21 protein levels • Reduced SA-β-gal activity • Reduced IL-8, IL-1α, and IL-1β mRNA levels	^202^
	Lifelong 40% caloric restriction	Age-related mitochondrial dysfunction	24-month-old B6D2F1 mice isolated mitochondria and permeabilized fibers from gastrocnemius muscle	• Increased respiratory function • Increased mitochondrial efficiency • Decreased uncoupling • No change in mitochondrial abundance or biogenesis	^206^
	60% caloric restriction from 14 weeks of age	Age-related mitochondrial dysfunction	22-24-month-old C57BL/6 mice adipose-derived stem cells	• Reduced mitochondrial activity • Reduced SA-β-gal activity	^207^
	Mitochondrial transplantation from mesenchymal stem cells to senescent cells	Senescence-associated mitochondrial dysfunction	Replicative senescent human retinal pigment epithelial cells (ARPE-19)	• Decreased mitochondrial and intracellular ROS • Increased mitochondrial fission markers • Increase in Parkin and decrease in PINK1 protein levels • Reduced p16 and p21 protein levels • Reduced SA-β-gal activity • Reduced cell size • Reduced NF-κB phosphorylation • Reduced IL-8 and TNF-α mRNA levels	^208^
	CCCP-induced Parkin-mediated mitochondrial clearance	Elimination of mitochondria from the cell	Irradiation-induced senescent human fetal lung fibroblasts (MRC-5)	• Reduced p16 and p21 protein levels • Reduced SA-β-gal activity • Reduced cell size • Reduced ROS • Reduced senescence-associated heterochromatin foci • Reduced SASP factor secretion (IL-6, IL-8, GRO and MCP-1) • Decreased mTOR activity • Initial small increase in proliferation followed by proliferative arrest	^98^
DAMP release	CRISPR-Cas9 deletion of BAX and BAK macropores	Blocking mtDNA release	Irradiation-induced senescent BAX^−/−^BAK^−/−^ human fetal lung fibroblasts	• Reduced expression of SASP factors • No impact on other senescence markers (p16, p21, SA-β-gal activity, γH2A.X foci, Lamin B1, HMGB1, or proliferation)	^183^
	BAX channel inhibitor BAI1	Blocking miMOMP-mediated mtDNA release	Irradiation-induced senescent human fetal lung fibroblasts (MRC-5 and IMR-90)	• Reduced mtDNA release • Reduced IL-6, IL-8, and IL-1β expression	^183^
			23-month-old C57BL/6 mice	• Reduced frailty score • Improved neuromuscular coordination • Improved grip strength • Improved spine and femur trabecular bone microarchitecture • In bone, reduced SASP factor expression with no change in p16, p21, or p53 • In brain, reduced p16-positive cells • Decrease in the senescence gene panel SenMayo^374^ in microglia and oligodendrocytes	^183^

### OXPHOS dysfunction and ROS generation

The generation of ATP through oxidative phosphorylation (OXPHOS) is dependent upon a negative mitochondrial membrane potential (ΔΨm) between the mitochondrial intermembrane space and matrix established by the electron transport chain (ETC). The ETC is made up of four protein complexes (complex I–IV), which pump protons as they are reduced by electrons from NADH and FADH_2_ generated in the tricarboxylic acid (TCA) cycle. This proton gradient provides the energetic basis needed to catalyze the formation of ATP from ADP mediated by ATP synthase. The loss of ΔΨm emerges as a unifying theme across both cellular senescence and aging. Cells induced to senesce by a wide variety of stimuli undergo a decrease in ΔΨm^54–57^, which coincides with an increase in proton leakage across the inner mitochondrial membrane^57^. These changes are caused in part by an increase in mitochondrial uncoupling via upregulation of mitochondrial uncoupling protein 2^55,57–59^, which dissipates the proton gradient and reduces OXPHOS^60^. Mitochondrial uncoupling may initially be in response to an increase in ROS production^60^; however, chronic uncoupling compromises respiratory chain function and causes ROS accumulation, which can drive senescent phenotypes^55,57,59,61–64^. This leads to damage of mitochondrial components, such as mtDNA^55^, mitochondrial proteins^65^, and lipids^66^, which can further exacerbate damage in a feed-forward loop. For example, senescent cells exhibit increased ROS-mediated protein modification, including to enzymes involved in mitochondrial protein quality control and the repair of oxidized proteins, such as methionine sulfoxide reductase A and B^65^. Additionally, peroxidation of lipids by ROS leads to the formation of 4-hydroxy-2-nonenal (HNE), which can react with proteins to form adducts^67^; an increase in HNE-modified proteins is found in senescent cells, with many of the modified proteins localized to the mitochondria^65^. Misfolded or oxidized mitochondrial proteins—especially those involved in protein quality control—can result in further impairment of ETC complexes, which disrupt electron flow and increase electron leakage to generate ROS, which could abet the formation and persistence of further oxidative damage. In particular, ROS-mediated DNA damage has been implicated in senescence (see Box 1).

Multiple studies have shown that inhibition of ETC enzymes leads to senescence. Genetic or pharmacological disruption of complex I^56,61^, complex II^68^, and complex III^56,69^ induces senescence, and complex IV deficiency is a feature of senescent cells^70,71^. These interventions are accompanied by increases in ROS and decreases in ATP production and ΔΨm. Similarly, disruption of other mitochondrial enzymes that regulate TCA activity also induces senescence, even in the absence of DNA damage^72–74^. Interestingly, while chronic ETC inhibition promotes senescence, transient inhibition of complex III may paradoxically improve mitochondrial function^75^, perhaps through hormesis. OXPHOS dysfunction further promotes senescence by activating the SASP. Reduced OXPHOS gene expression and loss of ΔΨm increase ROS, which activate c-Jun N-terminal kinase (JNK) and promote formation of cytoplasmic chromatin fragments (CCFs)^76^. CCFs trigger cyclic GMP-AMP synthase - stimulator of interferon genes (cGAS-STING) signaling, amplifying inflammatory SASP factors and reinforcing senescence in neighboring cells^77–80^.

Interventions that act to either increase mitochondrial ROS or decrease ROS-protective mechanisms can induce senescence. For example, depletion of key antioxidant enzymes, including superoxide dismutase (SOD) 1^81^, SOD2^82^, catalase^83^, and glutathione^84,85^ promotes senescence. Administration of the cytokine and SASP factor transforming growth factor-β (TGF-β) in vitro to corneal endothelium cells decreases SOD2 expression, which increases ROS and senescence markers, including p16, p21, and SA-β-gal^86^. Conversely, other studies have demonstrated that increasing ROS defenses delays senescence. Mitoquinone mesylate (MitoQ) is a mitochondrial antioxidant, which can prevent induction of senescent markers in doxorubicin-induced human endothelial cells^87^. Overexpression of SOD3 in fibroblasts also increases replicative lifespan under both normoxic and hyperoxic conditions^88^. Finally, pretreatment of MRC-5 fibroblasts with the free radical scavenger α-phenyl-t-butyl-nitrone extends replicative lifespan^89^. Other antioxidants have also been shown to extend replicative lifespan, including methylene blue (MB)^90^, carnosine^91^, aminoguanidine^92,93^, and the NAD^+^ precursor nicotinamide^94^, although not every antioxidant does so^95^. This suggests that a reduction in ROS per se may not be solely responsible for extension in replicative lifespan but is likely due to additional mechanisms of action beyond antioxidant defense.

Box 1 ROS-mediated DNA damage in cellular senescence DNA damage is one of the primary drivers of cellular senescence; for example, DNA damage at telomeres, the protective ends of chromosomes, has been shown to drive senescence^55,375^. Telomeric DNA is rich in guanine^376^, the base most susceptible to oxidative damage, forming 8-oxoguanine DNA lesions^377^, making telomeres especially vulnerable to ROS-induced damage^375,377–379^. Induction of a single 8-oxoguanine lesion at a telomere is sufficient to induce senescence by blocking replication, leading to replication stress, DDR signaling, and ultimately cell cycle arrest via activation of p53^380^. 8-oxoguanine lesions also interfere with binding by telomere repeat binding factors 1 and 2 (TRF1 and TRF2), part of the shelterin complex that prevents telomeres from being recognized as double-strand breaks^381^. Dysregulation of the shelterin components themselves can also induce senescence: loss of TRF2 induces senescence in vitro^382,383^ and in vivo^383^, downregulation of TPP1 promotes telomere damage and senescence^384^, and shelterin itself can interfere with DNA damage repair^385^ and contribute to persistent DNA damage^386,387^.

Damage to mtDNA can also induce cellular senescence. Mice expressing a defective form of the mtDNA polymerase gamma (POLG) exhibit increased mtDNA mutation burden, progeria phenotypes, and increased senescent burden^149,388^, suggesting that mtDNA damage can induce senescence. Suggestively, POLG mutant mice also exhibit an increase in oxidative damage in muscle tissue^389^ and in mtDNA and telomeric DNA^390^. However, whether mtDNA damage occurs at sufficient levels to cause senescence during normal aging has not been determined. Increased levels of mtDNA have been observed in RS human fibroblasts^55^, although whether this is causative of senescence or merely correlative was not established and remains an important question.

ROS also functions as a signaling molecule to reinforce senescence. Following DNA damage, a signaling cascade occurs through progressive phosphorylation and activation of ATM, AKT, and mammalian target of rapamycin complex 1 (mTORC1). mTORC1 activation induces expression of PGC-1β, which promotes mitochondrial biogenesis and increases ROS production. Elevated ROS levels, in turn, cause DNA damage and create a persistent DDR that sustains proliferative arrest^98^. mTORC1 also plays a role in regulating the SASP through downstream NF-κB^391^ and MAP kinase-activated protein kinase 2 (MAPKAPK2) activation^392^. Consequently, mTORC1 inhibition using rapamycin reduces secretion of several SASP factors^98^. Lastly, effectors of the DDR including p16^64^, p21^57,393^, and p53^394^ also promote ROS production when activated, supporting a positive feedback loop that maintains senescence.

### Mitochondrial elongation and impaired mitophagy

Senescent cells may compensate for functional defects in mitochondria by increasing mitochondrial biogenesis. Increased mitochondrial mass in senescent cells is observed in vitro^55,96,97^ as well as in vivo^98^. This is the result of increased expression of a number of proteins that are responsible for mitochondrial biogenesis, including peroxisome proliferator-activated receptor gamma coactivator 1-alpha (PGC-1α), PGC-1β, mitochondrial transcription factor A (TFAM), and nuclear factor erythroid 2-related factor 2 (NRF2)^56,96^. A reduction in mitochondrial turnover further contributes to increases in mitochondrial mass and dysfunction^99^. For example, basal levels of mitophagy – a lysosome-mediated degradation of dysfunctional mitochondria^100^ – driven by the PTEN-induced kinase 1 (PINK1)/Parkin/p62 pathway are reduced within two hours of SIS induction, suggesting impairment in mitophagy occurs early in senescence^101^. Several mechanisms likely drive this impairment. First, separating a dysfunctional mitochondrion from the mitochondrial network through fission is a prerequisite for mitophagy^102,103^. In senescent cells, mitochondria display an elongated and interconnected morphology that coincides with a downregulation in expression of the fission machinery dynamin-related protein 1 (DRP1) and mitochondrial fission 1 protein (FIS1)^104^. p53 also plays a large role in altering mitochondrial dynamics and impairing mitophagy in senescence (see Box 2). In addition, a decrease in S-nitrosoglutathione reductase activity in senescent cells causes excessive S-nitrosylation of Parkin, inhibiting its activity and thus hindering mitophagy^105^. Finally, elevated mTORC1 activity results in reduced capacity for lysosomal degradation^106,107^. Altogether, these changes reduce mitophagy, which results in accumulation of lower quality mitochondria, ultimately increasing ROS production^56,98^.

A growing body of evidence has demonstrated that direct impairment of mitophagy induces senescence^108–110^, while activation of mitophagy can mitigate senescence^111–114^, revealing mitophagy as a potential therapeutic target for senescence. In lung fibroblasts, treatment with a mitophagy inhibitor causes mitochondrial elongation, increased mitochondrial mass, and increased senescence markers^106^. Conversely, activation of mitophagy using small molecules reduces senescence markers in SIS human dermal fibroblasts, although this does not affect the SASP factors IL-6 and IL-8^101^. Similarly, insulin-like growth factor 1 (IGF-1) suppresses RS in smooth muscle cells, an effect dependent on the activity of PINK1^115^. In mice, PINK1 knockout leads to mitochondrial dysfunction, increased cellular senescence, and elevated SASP in aging renal cells^116^. Impaired mitochondrial dynamics and quality control may also promote inflammation, as mitochondrial elongation facilitates NLR family pyrin domain-containing 3 (NLRP3) inflammasome assembly^117^. NLRP3 inflammasome activation also occurs via the generation of ROS through complex I or III inhibition or impaired mitophagy^118,119^. Additionally, mitophagy impairment increases ROS production, which activates JNK signaling to promote inflammation and senescence^120^. In summary, impaired mitochondrial quality control produces dysfunctional mitochondria which induces senescence via ROS-mediated DNA damage and supports inflammation through NLRP3 inflammasome and JNK activation.

Changes in mitochondrial morphology also directly influence senescence. Overexpression of PGC-1 in human fibroblasts increases mitochondrial mass causing increased SA-β-gal activity and slowed cellular proliferation^121^. This apparent pro-senescent effect is likely due to elevated ROS production rather than greater mitochondrial mass per se, as a separate study found that treatment with resveratrol and salidroside suppress senescence by increasing mitochondrial biogenesis without increasing ROS^122^. In addition to changes in mitochondrial mass, inducing an elongated mitochondrial morphology by knockdown of FIS1 also leads to cells with a senescent morphology and increased SA-β-gal activity; conversely, reintroducing FIS1 reduces SA–β-gal activity^123^. Furthermore, knockdown of OPA1 causes mitochondrial fragmentation and reduces senescent phenotypes in FIS1-knockdown cells^123^. Promotion of mitochondrial elongation by targeting other fission or fusion regulators, such as downregulation of DRP1^124,125^ or overactivation of mitofusin 1 (MFN1)^126^, can also induce senescence. Altogether, these findings support a causal role for disrupted mitochondrial dynamics and impaired mitophagy in the induction of senescence.

Emerging evidence suggests that interactions between mitochondria and the endoplasmic reticulum (ER) play a role in determining mitochondrial dynamics in senescence. Mitochondria-ER contact sites (MERCS) help mediate a wide variety of processes, including calcium handling, lipid synthesis and trafficking, autophagy, and inflammation^127^. It is known that the number of MERCS generally increases in senescence, and artificial linkage of the mitochondria to ER promotes senescence^128,129^. MERCS also act as regulators of mitochondrial dynamics by localizing fusion and fission machinery^130,131^. A recent study found that in senescent cardiomyocytes, the senolytic quercetin inhibits cellular senescence by preserving MERCS, which improves mitochondrial morphology and function^132^, suggestive of a link between MERCS, mitochondrial dynamics, and senescence.

Box 2 Role of p53-mediated mitochondrial quality control in cellular senescence Investigations into the molecular mechanisms linking cellular senescence to alterations in mitochondrial quality control and morphology have focused on p53, which both inhibits fission and mitophagy. p53 increases the inhibitory phosphorylation of DRP1 by activating protein kinase A, causing suppression of mitochondrial fission and promotion of mitochondrial elongation, which decreases ΔΨm, and increases ROS production^395,396^. p53 also binds to Parkin to prevent its translocation to damaged mitochondria, inhibiting mitophagy, decreasing ΔΨm, and increasing ROS production^106,397,398^. Conversely, p53 knockdown reduces mitochondrial length by altering optic atrophy 1 (OPA1), a protein involved in mitochondrial fusion, and DRP1 expression, which improves mitophagy and reduces mitochondrial ROS^373^.

### Mitochondrial calcium accumulation

Altered calcium handling is another characteristic of mitochondrial dysfunction in senescence in which MERCS play a role. Inositol 1,4,5-trisphosphate receptor, type 2 (ITPR2) is a calcium channel present in the ER and at MERCS responsible for the movement of calcium into the cytosol. At MERCS, ITPR2-mediated calcium release allows for uptake into the mitochondria via the mitochondrial calcium uniporter (MCU). During OIS and RS, increased calcium release via ITPR2, combined with MCU-mediated mitochondrial uptake, causes calcium accumulation in the mitochondrial matrix, reducing ΔΨm and elevating ROS production^133^. Notably, treating cells under oxidative stress with a calcium chelator prevents senescence^134^. Similarly, knockdown of ITPR2 prevents the establishment of senescence in response to oncogenic stress, and activation of ITPR2 induces expression of senescence markers^133^. Forcing the formation of MERCS via expression of mitochondria-ER linkers can also promote senescence through the accumulation of calcium in the ER^135^. Conversely, knockout of *Itpr2* in mouse embryonic fibroblasts reduces the number of MERCS, thereby decreasing calcium flux and mitochondrial calcium accumulation and ultimately limiting senescence induction^135^.

Calcium accumulation activates the mitochondrial permeability transition pore (mPTP), a nonselective, voltage-gated channel located in the inner mitochondrial membrane that opens in response to elevated calcium in the matrix^136^. The consequences of pore opening depend upon its duration, where sustained activation leads to the collapse of ΔΨm, loss of ATP production, and ultimately cell death^137^. Activation of the mPTP increases with age across various cell types^138^, is triggered by ROS, and is capable of promoting further ROS production^139^. Senescent cells exhibit increased mPTP activity^140,141^, although current evidence suggests this may function as a compensatory mechanism to support cell survival rather than a driver of senescence. Cyclophilin D (CypD), the primary positive regulator of mPTP opening, was recently identified as a senolytic target^141^. Inhibiting CypD in senescent cells leads to mitochondrial calcium accumulation and cell death. Notably, senescent cells display frequent transient opening of the mPTP, a phenomenon termed mPTP “flickering”, which serves as a mechanism to offload excessive calcium into the cytosol, thereby preventing apoptosis. Conversely, sustained mPTP activation has also been shown to function as a senolytic^142^. Together, these findings suggest that senescent cells regulate mPTP dynamics in an attempt to balance calcium homeostasis and ensure survival, making the mPTP an important target for future development of senolytics.

### Metabolic changes

As key regulators of cellular metabolism, mitochondria are both affected by and contribute to the diverse array of metabolic changes that occur during senescence^143^. Mitochondria play important roles in regulating the levels of nicotinamide adenine dinucleotide (NAD), generating its reduced form (NADH) during the citric acid cycle and subsequently its oxidized form (NAD^+^) during OXPHOS. NAD serves as a cofactor in numerous metabolic reactions; for example, malate dehydrogenase utilizes the reduction of NAD^+^ to NADH to catalyze the reversible conversion of oxaloacetate to malate. In senescent cells, the activity and protein level of cytosolic malate dehydrogenase 1 (MDH1) is reduced, which leads to decreased levels of NAD^+^^144^. During senescence, reduced activity of nicotinamide phosphoribosyltransferase (NAMPT), the rate-limiting enzyme in NAD^+^ salvage, further decreases NAD^+^ levels^145,146^. Compounding this, opening of the mPTP facilitates the loss of NAD^+^ from the matrix, where it is subsequently hydrolyzed by the glycohydrolase CD38 in the intermembrane space^138,147^.

Altogether, these processes result in a decreased NAD^+^/NADH ratio. NAD^+^ is necessary for glycolysis, where it is utilized by glyceraldehyde-3-phosphate dehydrogenase (GAPDH) in the conversion of glyceraldehyde 3-phosphate to 1,3-bisphosphoglyceric acid. Thus, reduced NAD^+^/NADH inhibits glycolysis and ATP production^148^, leading to an increased AMP + ADP/ATP ratio^149,150^. Decreases in the efficiency of the ETC in senescent cells^57,59^ also decrease production of ATP, further increasing the ratio. An increased AMP + ADP/ATP ratio then activates the energy sensor and regulator AMP-activated protein kinase (AMPK)^151^. AMPK activation in turn induces mitochondrial biogenesis through direct phosphorylation of PGC-1α^152^, as well as inducing epigenetic changes that increase transcription of genes involved in mitochondrial biogenesis^153,154^.

The reduction in the NAD^+^/NADH ratio and increase in the AMP + ADP/ATP ratio act to reinforce and promote senescent phenotypes. NAD^+^ is used in a number of DNA repair systems, including poly-ADP ribose polymerases (PARPs) and sirtuins^155^. PARP1 inhibition in irradiated cancer cells facilitates the persistence of DNA damage foci and thereby promotes senescence^156^. Similarly, inhibition of sirtuins can promote senescence as these NAD^+^-dependent histone deacetylases mediate access of DNA repair machinery to sites of DNA damage^157^. In addition, sirtuin activity limits the SASP by deacetylation of the promoter regions of IL-6 and IL-8 and repressing NF-κB activation^158,159^. The mitochondrial sirtuin SIRT3 also plays a role in inhibiting mPTP opening by deacetylating CypD^160^. Direct modulation of enzymes that regulate the NAD^+^/NADH ratio can impact senescence. For example, reduction of the cytosolic NAD^+^/NADH ratio via knockdown of MDH1 induces senescence^144^. Interestingly, knockdown of the mitochondrial malate dehydrogenase 2 (MDH2), which affects mitochondrial–but not cytosolic—NAD^+^ levels, does not affect cellular senescence in young human dermal fibroblasts^144^. Similarly, inhibiting the shuttling of NADH produced during glycolysis to mitochondria by the malate-aspartate shuttle with aminooxyacetate, which lowers cytosolic but not mitochondrial NAD^+^/NADH ratios, causes senescence^149^. These data suggest that cytosolic, but not mitochondrial, NAD^+^ levels impact senescence induction.

These findings are not limited to NAD^+^; increasing the AMP + ADP/ATP ratio of a cell by the addition of exogenous AMP also induces senescence^161^. A consequence of an increased AMP + ADP/ATP ratio is the activation of AMPK, which directly phosphorylates p53, promoting p21 transcription and cell cycle arrest^162^. Activated AMPK also prevents the translocation of the mRNA-stabilizing protein human antigen R (HuR) from the nucleus to the cytosol^163^. HuR binds to and stabilizes mRNA of proliferative genes c-Fos, cyclin A, and cyclin B; thus, its reduced presence in the cytosol reduces translation of these genes and aids proliferative arrest^164^. Interestingly, in OIS, but not RS, an increase in NAMPT expression increases the NAD^+^/NADH ratio, which suppresses AMPK activity^150^. This reduces p53 signaling and leads to enhanced activation of NF-κB through increased p38 mitogen-activated protein kinase (MAPK) signaling, promoting the SASP^150^. These findings suggest that the role of AMPK in senescence is context-dependent and influenced by the mode of senescence induction. Lastly, multiple studies have demonstrated that activation of AMPK through small molecules or by genetic means induces senescence^162,163,165,166^; however, this is context dependent, as AMPK activation can also suppress senescence, particularly in SIS models^167–170^. Altogether, these studies highlight the significance of metabolic changes in driving senescence, although it is important to recognize that this is not solely limited to the changes in NAD^+^/NADH or AMP + ADP/ATP ratios described here. Fatty acid oxidation (FAO), for example, has been implicated in senescence, discussed in Box 3.

Box 3 FAO in senescence During FAO, lipids are catabolized into acetyl-CoA, producing NADH and FADH_2_ in the process. Acetyl-CoA is then used in the TCA cycle, ultimately producing ATP through OXPHOS. This process occurs largely in the mitochondrial matrix^399^. FAO has been implicated in senescence. Pharmacological activation of FAO increases mitochondrial ROS production, decreases ΔΨm, and induces senescence in lung fibroblasts^400^. This is mediated through DNA-damage induced activation of ATM, which translocates to the outer mitochondrial membrane where it phosphorylates a transmembrane protein Bcl-2 interacting protein 3 (BNIP3). Activated BNIP3 increases mitochondrial cristae, facilitating an increase in FAO. FAO supplies acetyl-CoA, which mediates an increase in histone acetylation to drive p16 expression and proliferative arrest^400^. While the mechanism by which FAO induces senescence was not elucidated, ROS-induced DNA damage is a probable candidate. This study replicates previous findings made in OIS^401^ and RS endothelial cells, which also found a role for FAO in the SASP^402^. However, this is in conflict with several studies which describe the opposite, a decrease in FAO, in different contexts, including irradiation-induced senescent hepatocytes and fibroblasts^403^ and oxidative-damage induced senescent endothelial cells^404^. Thus, the role of FAO in senescence likely varies substantially depending upon the cell type and mechanism of senescence induction.

### Release of damage-associated molecular patterns (DAMPs)

Certain mitochondrial components can act as damage-associated molecular patterns (DAMPs), which elicit an inflammatory response when present in the cytoplasm or extracellular space. This is likely due to the bacterial origin of mitochondria, as many DAMPs contain elements that are characteristic of bacteria^171^. DAMPs are recognized by immune cells via pattern recognition receptors, inducing an immune response. Some examples of DAMPs are mtDNA^172,173^; cardiolipin, an inner mitochondrial membrane lipid^174^; and TFAM^175,176^. Aspects of mitochondrial dysfunction observed in senescent cells, such as ΔΨm depolarization and impairment in mitophagy, likely result in increased release of DAMPs^176,177^.

mtDNA has received the most attention in its capacity as a DAMP to drive the SASP. Like bacterial DNA, mtDNA contains unmethylated CpG islands, which are recognized by toll-like receptor 9 (TLR9) in endosomes^178^, initiating a signaling cascade that causes inflammation by activation of p38 MAPK and increased expression of NF-κB^172,173,179,180^. Cytoplasmic mtDNA is also detected by cGAS, which catalyzes the formation of the second messenger cyclic GMP-AMP dinucleotide (cGAMP) and activates STING, culminating in the expression of type I interferons^181^. Circulating mtDNA in serum is known to both increase with age and correlate with levels of inflammatory cytokines^182^, and an inducer of senescence, cigarette smoke, causes release of mtDNA^106^. A recent study elucidated the mechanism by which mtDNA is released in senescence^183^. Mitochondria in senescent cells were found to undergo low-grade apoptotic stress, which, although insufficient to induce apoptosis, does cause limited mitochondrial outer membrane permeabilization (MOMP) termed minority MOMP (miMOMP). This facilitates the release of mtDNA into the cytosol through BAX/BAK channel pores, activating the cGAS-STING pathway and driving the SASP, but not affecting other senescence markers. Blocking mtDNA release by treating mice with a BAX inhibitor reduced SASP factor expression in the bone and improved measures of healthspan, with no impact on lifespan. Interestingly, miMOMP only occurs at fragmented mitochondria, suggesting mitochondrial elongation during senescence may be a means of decreasing miMOMP. Accordingly, promoting mitochondrial fusion does indeed reduce miMOMP^183^.

Aside from cGAS-STING, mtDNA also activates additional inflammatory pathways. Oxidized mtDNA can bind to and activate the NLRP3 inflammasome^184^, promoting IL-1β secretion^185^ and downstream NF-κB activation^186^. Interestingly, activation of NLRP3 requires mtDNA synthesis^118,187^, supporting a role for mitochondrial dysfunction in inflammasome engagement. Other mitochondrial DAMPs may also contribute to SASP release. Cardiolipin accumulates in senescent cells and can induce senescence^188^, in addition to binding to and activating the NLRP3 inflammasome^174^. Extracellular TFAM is also proinflammatory^189,190^. When bound to mtDNA, TFAM interacts with the membrane-bound receptor for advanced glycation end-products (RAGE) of plasmacytoid dendritic cells^191^. This facilitates the internalization of mtDNA and subsequent recognition by TLR9, resulting in expression of inflammatory factors. While suggestive, more work is needed to fully elucidate the extent different DAMPs contribute to the senescent phenotype.

### Direct mitochondrial manipulations reveal causality in cellular senescence

Certain interventions tested predominately in mouse and in vitro models broadly enhance mitochondrial function to delay the onset of senescence. Methylene blue (MB), a tricyclic phenothiazine drug historically used to treat malaria and methemoglobinemia^192^, acts as a catalytic redox cycler. MB accepts electrons from complex I, forming LeucoMB, which donates electrons to complex IV, regenerating MB^193^. This redox cycling enhances complex IV activity and may protect against oxidative damage by outcompeting oxygen for electrons^193,194^. MB also increases complex IV expression, elevates the NAD^+^/NADH ratio^194,195^, improves ΔΨm, reduces lipid peroxidation, and increases ATP production in rodent models of cholestatic disease and multiple sclerosis^196,197^. Notably, MB extends the replicative lifespan of normal human fibroblasts^194,195^ and reduces SA-β-gal activity and p16 levels in aged primary human fibroblasts^90^, suggesting that improved mitochondrial function may be delaying the onset of senescence.

Klotho is a protein present in both transmembrane and secreted forms that modulates oxidative stress, growth factor signaling, and ion transport^198^. Reduced Klotho levels have been implicated in senescence^199,200^. In a mouse model of kidney disease, ectopic Klotho expression lowers ROS, increases mitochondrial mass, and improves mitochondrial morphology^201^. These mitochondrial improvements were accompanied by a reduction in senescence markers, including SA–β-gal, p16 mRNA and protein levels, and γH2AX foci in renal tubular epithelial cells. In human cortical brain organoids, extended culture causes downregulation of Klotho and coincides with increases in p16 and p21 mRNA levels and SA-β-gal activity^202^. Upregulation of Klotho reduces these markers of senescence and expression of several SASP factors.

Caloric restriction (CR), one of the most well-established methods of increasing lifespan in a diverse variety of organisms^203^, reduces senescent cell markers in mice^204^, as well as humans^205^. Evidence suggests that this occurs at least in part through alteration in mitochondrial function. In aged mouse skeletal muscle, CR decreases ROS production, increases antioxidant scavenging, and reduces oxidative damage^206^. Similarly, CR reduces mitochondrial activity in mouse subcutaneous adipose-derived stem cells, which correlates with a decrease in SA-β-gal, suggesting a link between mitochondrial function and senescence^207^.

Due to the multifaceted mechanisms by which these interventions enhance mitochondrial function and the potential for other affects on the cell, it is difficult to definitively prove causality between improved mitochondrial function and reduced senescence in these instances. A direct test of causality comes from a study in which healthy mitochondria were transplanted into senescent cells^208^. The mitochondria of RS human retinal pigment epithelial cells produce more ROS, have impaired mitophagy, and altered DRP1 and MFN1 levels consistent with elongation compared to proliferating cells, indicative of mitochondrial dysfunction. Transplantation of mitochondria from human umbilical cord-derived mesenchymal stem cells into these senescent cells reversed mitochondrial dysfunction and reduced p16 and p21 levels as well as the SASP factors TNF-α and IL-8, providing strong evidence that mitochondrial dysfunction is a significant driver of cellular senescence.

Another direct test of the role of mitochondria in senescence involves complete mitochondrial depletion. This can be achieved through administration of the mitochondrial uncoupler carbonyl cyanide m-chlorophenylhydrazone (CCCP), which targets mitochondria for degradation via Parkin-mediated mitophagy^98^. In irradiation-induced senescent human fibroblasts, CCCP treatment effectively depletes mitochondria and causes a significant reduction in the senescent markers p16, p21, SA-β-gal, and senescence-associated heterochromatin foci, as well as a reduction in cell size and ROS production^98^. SASP factor secretion, specifically IL-6, IL-8, GRO, and MCP-1, was nearly abolished in these cells. Similar effects were found in RS, OIS, and oxidative-damaged induced senescent fibroblasts. Mitochondria-depleted cells continued to proliferate, albeit at a greatly reduced rate, until twenty days post-irradiation. Once arrested, senescence was not accompanied by the SASP. Thus, mitochondria appear to be central to the process of cellular senescence, particularly its inflammatory qualities.

## Mitochondrial dysfunction links neurodegenerative disease to cellular senescence

**Fig. 2:**
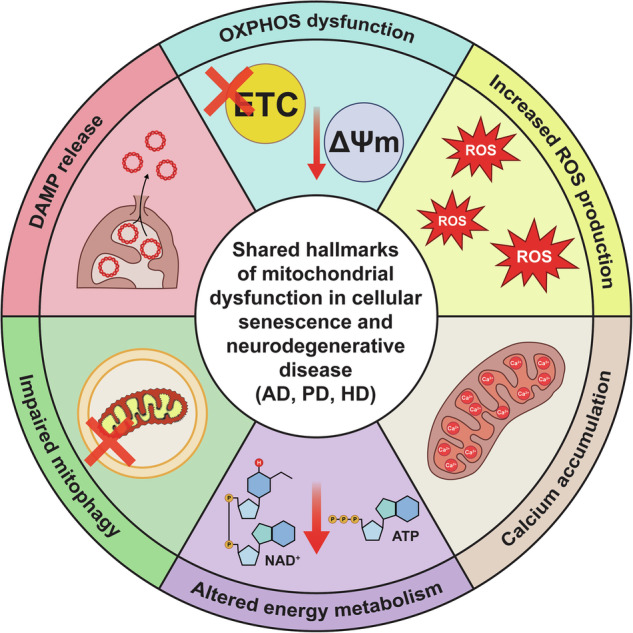
Mitochondrial dysfunction is a shared feature of cellular senescence and neurodegenerative disease. The mitochondria of senescent cells and neural cells in the context of neurodegeneration exhibit a wide array of commonalities, including OXPHOS dysfunction leading to a loss of mitochondrial membrane potential (ΔΨm), increased ROS production, and altered energy metabolism characterized by reduced NAD^+^ and ATP levels. Mitochondrial calcium accumulation further exacerbates these changes. Mitophagy is also impaired which reduces mitochondrial quality. Lastly, damage-associated molecular pattern (DAMP) release promotes inflammation. These many shared features suggest mitochondrial dysfunction in neurodegenerative disease may be inducing senescence to further drive neurodegeneration.

Aging is the greatest risk factor for neurodegenerative disease^209^, suggesting that fundamental biological hallmarks of aging, including mitochondrial dysfunction and cellular senescence, may also be drivers of neurodegenerative disease. Mitochondrial dysfunction is a hallmark of both inherited and sporadic neurodegenerative diseases^210^, and cellular senescence is similarly implicated in their progression^211,212^. These findings support a potential mechanistic link in which mitochondrial dysfunction promotes senescence, which in turn contributes to neurodegeneration. Notably, many of the same defects in mitochondria that occur in senescence are also observed in neurodegenerative disorders (Fig. 2).

Neurodegeneration is characterized by the progressive loss of neurons in the brain, which leads to impaired cognitive and physiological function^213^. Many common neurodegenerative disorders, including Alzheimer’s disease (AD), Parkinson’s disease (PD), and Huntington’s disease (HD) involve the accumulation of misfolded and aggregated proteins that disrupt mitochondrial function^214^. Given the brain’s exceptionally high metabolic demand^215^, neuronal health is particularly sensitive to these changes. In the following sections, we examine mitochondrial dysfunction and cellular senescence in AD, PD, and HD, focusing on shared pathology between aging, senescence, and neurodegenerative disease.

### Mitochondrial dysfunction and cellular senescence in Alzheimer’s disease

Mitochondrial dysfunction is a well-described feature of AD^216–219^ and has even been proposed as one of its primary drivers^217,220^. Many of the same features of mitochondrial dysfunction that occur in senescent cells are observed in AD contexts, suggesting overlapping mechanisms. Some of the hallmark pathologies of AD—including β-amyloid (Aβ) plaques and intercellular phosphorylated tau tangles—directly contribute to mitochondrial impairment and neurodegeneration^221^. For example, Aβ^222^ and certain forms of phosphorylated tau^223^ are taken up by mitochondria, where they negatively interact with ETC complexes. Exposure to Aβ significantly reduces complex I and IV activity in neurons^224,225^, likely due to ROS-induced mitochondrial membrane peroxidation^225^, while tau impairs complex IV activity in the P301L transgenic tau mouse model^226^. These alterations decrease ΔΨm and increase ROS production^214,225–228^, driving oxidative damage to both nuclear and mtDNA^229^.

Aβ also impairs calcium regulation^214,230,231^, which further amplifies mitochondrial dysfunction. In transgenic mice overexpressing human Aβ, mitochondrial calcium accumulation overload coincides with increased CypD expression and mPTP formation^232,233^. Aβ may promote this accumulation by increasing plasma membrane permeability and activating N-methyl-D-aspartate (NMDA) receptors^234^. Similar to what is found in senescent cells, MERCS are also altered in AD. AD brains exhibit a higher number of MERCS and shorter ER-mitochondria distances, which promote calcium accumulation and mPTP activation^235,236^. Importantly, reduction of MERCS in the 3xTg-AD mouse model, which develops both Aβ plaques and tau tangles, prevents mitochondrial calcium accumulation and ΔΨm depolarization in hippocampal neurons and reduces Aβ levels^237^, positioning MERCS as potential targets for future AD therapeutics.

As in senescence, OXPHOS dysfunction in AD disrupts cellular energetics. Decreased levels of ATP are found in cell and animal models of AD^224,226,238,239^, and dysregulation of AMPK activity has also been reported, although its precise role in AD progression, whether protective or detrimental, remains uncertain^240,241^. NAD^+^ depletion, a hallmark of brain aging and AD, further contributes to energetic failure; restoring NAD^+^ ameliorates pathology and improves cognition in several AD mouse models^242^. Increased CD38 activity may be partially responsible for this loss of NAD^+^, as its knockout or inhibition in the APP/PS1 amyloidogenic mouse model reduces Aβ levels in the brain and improves cognition^243,244^.

Alterations in mitochondrial morphology and quality control accompany these bioenergetic defects. For example, reduced NAD^+^ impairs sirtuin activity, compromising mitophagy in AD^242,245,246^. Mitophagy is also impaired in other ways by AD pathology^247,248^, including reduced recruitment of Parkin to PINK1-labeled mitochondria^249^. Unlike senescent cells, mitochondrial morphology in AD is typically characterized by fragmentation rather than elongation, as both Aβ^250^ and tau^251^ interact with DRP1 to promote mitochondrial fission. In addition, changes in expression of DRP1, FIS1, MFNs, and OPA1 in favor of fission occur in the AD brain^225,252^. However, some evidence suggests that mitochondrial elongation may occur, as tau disrupts the localization of DRP1 to mitochondria in a *Drosophila* model of AD^253^ and primary fibroblasts from AD patients exhibit mitochondrial elongation^254^. Unlike cellular senescence, mitochondrial biogenesis declines in AD, via decreased expression of PGC-1α^255,256^.

Mitochondrial-derived inflammatory signaling also contributes to AD pathology. Neuroinflammation is recognized as a prominent feature of AD^257,258^, and recent studies implicate mitochondrial DAMPs and the cGAS-STING pathway in this process. Cytosolic mtDNA levels are elevated in the brains of both the amyloidogenic 5×FAD mouse and naturally aged mice, and pharmacological inhibition of the cGAS-STING pathway reduces Aβ pathology in the 5×FAD model^259,260^. In the amyloidogenic APP/PS1 model, increased cytosolic mtDNA also triggers cGAS-STING activation, and a reduction in cytosolic mtDNA release by administration of melatonin or the NAD^+^ precursor nicotinamide riboside reduces inflammation^261^. Even in naturally aged mice, release of mtDNA into the cytosol of microglia similarly promotes cGAS-STING pathway signaling to drive microglia activation, inflammation, and neurodegeneration^262^, which can impair neurocognitive function in mice^263^. Overall, through their diverse means of inducing inflammation, mitochondrially-derived DAMPs are increasingly being recognized as players in AD and neurodegenerative disease more broadly^264–267^.

There exists substantial evidence linking cellular senescence to AD^268–270^, although much of it remains correlative or derived from aggressive transgenic mouse models. In the inferior parietal cortex of AD patients, up to 80% of large Aβ plaques are associated with p21-positive oligodendrocyte precursor cells^271^. In addition, neurons containing hyperphosphorylated tau neurofibrillary tangles exhibit a transcriptional profile characteristic of senescence, including upregulation of genes related to cell survival, stress response, and inflammation^272,273^. Consistently, neurons from AD prefrontal cortex display elevated senescence markers relative to age-matched controls^274^, and direct conversion of AD patient fibroblasts to neurons yields cells with senescence-like transcriptomic and epigenomic signatures and an inflammatory SASP capable of activating astrocytes^274^.

Single-nuclei transcriptomics and imaging mass cytometry further reveal that microglia are particularly susceptible to senesce^275^. Microglia Aβ load, proximity to Aβ plaques, and severity of AD all correlate with upregulation of genes associated with senescence. Significantly, microglia with Aβ load display a gene signature indicative of mitochondrial dysfunction, particularly loss of ΔΨm, suggesting Aβ may be inducing mitochondrial dysfunction and senescence in microglia^275^. Tau also mediates senescence in microglia. Co-culture of primary murine microglia with human tau monomers induces senescence and accompanying SASP^276^. Importantly, senescent microglia are functionally impaired, having a slower migration rate and reduced ability to clear tau.

Growing preclinical evidence supports senescent cells as a potential therapeutic target for AD. The senolytic cocktail dasatinib and quercetin (D + Q) is the most widely tested senolytic in both preclinical and clinical AD trials. D is a tyrosine kinase inhibitor FDA-approved to treat cancer, and Q is a flavonoid that, when combined with D, suppresses anti-apoptotic pathways in senescence to drive senolysis^277^. D is known to cross the blood-brain barrier (BBB) in humans^278^ while the pharmacokinetics of Q are less characterized in humans, although evidence in rats suggests it is also BBB permeable^279^. In a mouse model of Aβ accumulation, intermittent administration of D + Q for 11 weeks significantly reduced Aβ load and improved measures of memory^271^. In this model, p16 mRNA-positive cells were almost exclusively oligodendrocyte precursor cells localized to areas of Aβ deposition, suggesting their clearance mediated these positive effects. In the rTg(tauP301L)4510 mouse model of tauopathy, which develops aggressive tau pathology in the forebrain and consequently neurodegeneration, the forebrain exhibits increased DNA damage, p16 and p21 expression, and NF-κB activation^272^. Interestingly, decreased respiratory capacity was also described in the cortex and hippocampus, although no changes in mitochondrial mass were present. Intermittent treatment of these mice with D + Q decreased the expression of senescence genes and the number of tau neurofibrillary tangle-bearing cortical neurons as compared to vehicle-treated animals^272^. Similar findings were reported in the PS19 tauopathy model, which expresses mutant tau specifically in neurons, where p16-positive astrocytes and microglia accumulate in the hippocampus and cortex^280^. Genetic clearance of these cells prevented neuronal loss in the dentate gyrus and improved short-term memory. A more recent study which administered D + Q in PS19 mice at month 3 of age to month 9 validated these findings^281^. D + Q administration maintained BBB, attenuated hippocampal and neocortex brain atrophy, reduced hyperphosphorylated tau levels, and improved performance on a memory task. In addition, D + Q treatment shifted microglia from a disease-associated state to a homeostatic state^281^.

With growing preclinical evidence supporting a causal role for cellular senescence in AD, several Phase I clinical trials using D + Q in small patient populations with mild-cognitive impairment or AD have been completed^282–284^. D + Q was found to be safe with only a small incidence of mild adverse effects reported, and although the studies were not sufficiently powered to assess efficacy, results suggest possible decreases in inflammatory SASP factors, paving the way for future studies.

### Mitochondrial dysfunction and cellular senescence in Parkinson’s disease

Mitochondrial dysfunction has been strongly implicated in PD^285–288^, perhaps more than any other neurodegenerative disease, and shares many of the features observed in AD. PD is characterized by the accumulation of the protein α-synuclein (α-syn), encoded by *SNCA*, into Lewy body aggregates and the selective loss of dopaminergic neurons in the substantia nigra^289^. α-syn preferentially binds mitochondrial membranes through interactions with cardiolipin^290,291^, which may underlie the association between mitochondrial dysfunction and PD. ETC complexes, particularly complex I, are impaired in PD with decreases in both protein level and activity^292–294^. α-syn-mediated complex I dysfunction decreases ΔΨm and increases ROS production^214,227,228,295,296^, resulting in increased oxidative damage^297–299^, including damage to nuclear DNA^300^ and mtDNA^301^. α-syn also disrupts calcium homeostasis: the formation of calcium-permeable pores in cell membranes increases cytosolic calcium^302,303^, which promotes mitochondrial calcium overload and opening of the mPTP^214,231,295^. Dysregulation of MERCS by α-syn is reported, although conflicting evidence makes it difficult to determine whether α-syn promotes or impairs MERCS formation^288^.

As in senescence, ETC dysfunction in PD perturbs cellular metabolism. ATP production is reduced in the skeletal muscle of PD patients^304^, in cells exposed to α-syn^296,305^, and in a *Drosophila* model of PD^306^. Like AD, the role of AMPK in PD is controversial. Both an increase and a decrease in AMPK have been reported in PD. AMPK activation may have beneficial effects by inducing autophagy and promoting α-syn clearance, or may promote α-syn aggregation by mediating α-syn phosphorylation^240,307^. In addition to ATP, NAD^+^ levels are reduced in animal and cell models of PD^242^, and in skeletal muscle of PD patients^304^. A reduction in NAD^+^ may contribute to impaired mitophagy as sirtuins are known mitophagy regulators^308^ and NAD^+^ activates mitophagy in cell models of PD^309^. Mutations in mitophagy-regulating genes encoding PINK1 and Parkin are similarly implicated in familial forms of PD^310^. In addition, exposure to α-syn affects autophagy and mitophagy, typically increasing activity in an effort to clear α-syn and defective mitochondria^311,312^. Changes in mitophagy are also linked to mitochondrial fragmentation, another feature of PD^313^. Transfection of cells with α-syn causes changes in expression of DRP1 and OPA1 in favor of fission and mitochondrial fragmentation^311^, which may partially explain the increase in mitophagy observed in PD. Finally, like AD, and unlike cellular senescence, mitochondrial biogenesis declines in PD models^314^ and PGC-1α-mediated mitochondrial biogenesis displays neuroprotective effects in dopaminergic neurons of PD cell and mouse models^315,316^.

Neuroinflammation has long been recognized as a feature of PD^257,317^, with recent research beginning to implicate mitochondria as a source of inflammation. Mitochondrial complex I inhibition in microglia activates NLRP3 inflammasome activity and results in dopaminergic neuron loss^318^. In PD patients, circulating levels of mtDNA are higher^319^, and mtDNA-induced cGAS-STING pathway activity occurs in different PD mouse models^320–322^. Furthermore, activation of TLR9 signaling through administration of mtDNA to the substantia nigra of mice leads to activation of microglia and loss of dopaminergic neurons^323^. These results suggest that an investigation into the potential involvement of other mitochondrial-derived DAMPs in PD-associated neuroinflammation is warranted.

Although less extensively studied than AD, evidence also exists linking PD to senescence of neural cells^324^. In PD patient tissue, the substantia nigra displays increased expression of p16 and SASP factors as well as loss of Lamin B1 in astrocytes^325^. Treatment of cultured human astrocytes with paraquat, an herbicide associated with the onset of PD, induces senescence^325^. Likewise, administering mice paraquat induces senescence in astrocytes and causes phenotypic changes resembling PD, including loss of dopaminergic neurons in the substantia nigra and deficits in motor function; genetic ablation of p16 expressing cells ameliorates these effects^325^. Another study found signs of senescence in post-mortem midbrain tissue from PD patients, including reduced levels of HMGB1 and Lamin B1, along with an increase in p21, but not p16, expression^326^. α-syn preformed fibril treatment in isolated cortical neurons, astrocytes, and microglia also induces a senescent phenotype, although less dramatically in cortical neurons^326^. Injection of α-syn preformed fibrils into the striatum of mice decreased HMGB1 and Lamin B1 expression and increased p21 expression in astrocytes and microglia in the substantia nigra compacta, striatum, and cortex^326^. Overexpression of human α-syn in the brain of mice causes DNA damage^327^, suggesting this may be one of the mechanisms whereby it induces senescence. Mirroring findings made in mouse models, midbrain organoids produced from induced pluripotent stem cells (iPSCs) of patients with familial PD exhibit dopaminergic neuron loss and the presence of astrocyte senescence^328^. Providing evidence that mitochondrial dysfunction plays an important role in senescence development in PD, metformin was found to delay astrocyte senescence in a mouse model of PD^329^. Metformin elevated MFN2 expression, which decreased mtDNA release and reduced the SASP through decreased cGAS-STING signaling, ultimately preventing loss of dopaminergic neurons. As senescence data in PD patients remains correlative, clinical trials of senolytics are needed to robustly implicate senescence in PD.

### Mitochondrial dysfunction and cellular senescence in Huntington’s disease

HD is an inherited autosomal dominant condition caused by an expanded CAG nucleotide repeat of variable size in the *HTT* gene encoding the protein huntingtin. This mutant form of huntingtin (mHTT) contains polyglutamine repeats, which are prone to aggregation and cause neuronal dysfunction and death, particularly in the striatum^330^. As with AD and PD, mitochondrial dysfunction is a prominent feature of HD^331–333^. mHTT is produced within cells and interacts with mitochondria, particularly at the outer mitochondrial membrane, where it interferes with mitochondrial function^334,335^. In HD patients, the activity of ETC complexes II–IV is reduced in the caudate nucleus, an area of the brain that experiences significant neurodegeneration in HD^336^. This impairment in ETC complexes may explain the reduction of ΔΨm found in lymphoblasts of HD patients and in mouse models expressing mutant mHTT^334^. Mitochondrial ROS production is also higher in skin fibroblasts from HD patients and the striatum of the YAC128 mHTT-expressing HD mouse model^337^, which can drive oxidative damage^337–340^, including to nuclear DNA and mtDNA^341^. Impaired mitochondrial calcium handling is also characteristic of HD: medium spiny neurons in YAC128 mice are prone to calcium overload and subsequent mPTP opening^342^, potentially due to the action of mHTT, which has been demonstrated to lower the calcium threshold needed for mPTP opening^343^. The tendency for calcium overload may be caused by mHTT interacting with inositol 1,4,5-trisphosphate receptors (IP3R), calcium channels located in the ER, to increase their responsiveness and promote calcium uptake by mitochondria^344^.

Consistent with AD, PD, and cellular senescence, OXPHOS dysfunction in HD leads to deficits in energy metabolism. Decreased ATP levels are observed in both the YAC128^345^ and R6/2^346^ HD mouse models and in the cerebrum^347^ and skeletal muscle^348^ of human PD patients. In general, AMPK overactivation is observed in HD mouse models^240,349^, although activation of AMPK has also been reported as beneficial in HD models^350–352^, making it difficult to draw broad conclusions about its role in HD. In *Drosophila* HD models, depletion of NAD^+^ levels correlates with HD progression^353,354^, and supplementation with NAD^+^ precursors has shown therapeutic potential in preclinical models^242^. Beneficial effects of NAD^+^ supplementation may be due to SIRT1-PGC-1α-induced mitochondrial biogenesis^355^. Indeed, mHTT interferes with the PGC-1α promoter to repress its expression, likely impairing mitochondrial biogenesis^356^, and PGC-1α upregulation has therapeutic effects in mHTT-expressing mouse models^356,357^. Mitochondrial morphology is also impaired in HD, exhibiting fragmentation. HD patients display increased expression of DRP1 and FIS1 and decreased expression of MFN1, MFN2, and OPA1 in the striatum, consistent with mitochondrial fission^358^. In addition, mHTT interacts with DRP1 to enhance its enzymatic activity and promote mitochondrial fission^359^. Increased DRP1-mediated mitochondrial fission in primary striatal neurons of the R6/1 HD mouse model reduces MERCS and impairs mitochondrial calcium handling^360^. mHTT also inhibits mitophagy by interfering with the formation of complexes necessary for autophagy initiation and impairing mitophagy receptors^361^.

As with AD and PD, neuroinflammation characterizes HD^362^, with recent findings suggesting a potential role for mitochondrial DAMPs in promoting inflammation. cGAS, phosphorylated STING, and cGAS-dependent inflammatory genes are upregulated in striatal cells of HD patients^363^. In R6/2 mice, increased cytosolic mtDNA triggers cGAS-STING activation, and a reduction in cytosolic mtDNA release by administration of melatonin or the NAD^+^ precursor nicotinamide riboside reduces inflammation^364^. Less evidence exists for NLRP3 inflammasome activation in HD, although increased expression of NLRP3 in peripheral blood mononuclear cells of HD patients^365^ suggests further investigation is warranted.

The study of the role cellular senescence plays in HD is still in its infancy, although senescence has begun to be considered as a potential contributor^366^. Like AD and PD, models of HD exhibit an increase in senescent markers. In the striatum of HD knock-in mouse models, gene pathways associated with cellular proliferation, DDR, and senescence are differentially regulated^367^. iPSC-derived neural stem cells of HD patients showcase increased p16 expression and SA-β-gal activity; when these cells are differentiated into medium spiny neurons, markers of senescence remain, suggesting HD pathology may be driving senescence^368^. Levels of inflammatory cytokines and matrix metalloproteinases in the plasma of HD patients and R6/2 mice are elevated, including the prominent SASP factors IL-6 and MMP-9^369^. Senescence-focused interventions to target HD are still generally lacking. Fisetin, a flavonoid polyphenol and known senolytic agent^370^, suppresses HD pathology in a *Drosophila* and mouse model of HD^371^, although, since this study was performed before fisetin’s discovery as a senolytic, no analysis of a possible role for senescence was explored. The many shared hallmarks of mitochondrial dysfunction in both senescence and HD, as well as AD and PD, point to a common etiology. This suggests a fruitful line of research investigating whether the mitochondrial dysfunction characteristic of neurodegenerative disease induces senescence and subsequently targeting mitochondria as the nexus between cellular senescence and neurodegenerative disease.

## Conclusion

The accumulation of senescent cells with age is now recognized as a major contributor to age-related decline. Current evidence strongly supports the idea that mitochondrial dysfunction is not just a consequence of senescence, but a central driver of its onset and its associated proinflammatory phenotype. Key mitochondrial defects, including ETC dysfunction, increased ROS production, calcium accumulation, changes in energy metabolism, dysregulated mitochondrial dynamics, reduced mitochondrial quality control, and release of DAMPs are all known to play a role in senescence. Notably, these same mitochondrial abnormalities are also prominent features of neurodegenerative disease, suggesting a mechanistic link between mitochondrial dysfunction, the induction of senescence, and neurodegeneration.

This growing overlap highlights mitochondrial pathways as a promising target of both anti-senescence and neurodegeneration therapies. Indeed, evidence suggests that many current anti-senescence interventions exert their effects by modulating mitochondrial function. Deepening our understanding of how mitochondrial function is impaired in senescent cells and how impaired mitochondria in turn influence senescence will likely prove valuable for precise and effective anti-senescence therapies. Ultimately, targeting these mechanisms may yield broad benefits in combating both aging and neurodegenerative diseases.

## Data Availability

No datasets were generated or analyzed during the current study.
